# Allegheny County Veterinary Medical Association

**Published:** 1901-10

**Authors:** 


Secretary.

### ALLEGHENY COUNTY VETERINARY MEDICAL ASSOCIATION.

THE Allegheny County Veterinary Medical Association was organized on Thursday, August 22d, at the Hotel Schenley, and several representatives of the profession and the cause were there, among them Drs. Ardary, Boyd, Bachman, Gerhard, Gillmore, Laycock, Richards, and Spindler. Temporary officers were elected as follows; Chairman, Dr. S. J. Laycock; Secretary, Dr. Gerhard. The greatest interest was shown in the movement, and it promises to be a very successful organization. A permanent organization will be formed later, when a constitution and by-laws will be adopted.

---

At the opening of the McKillip Veterinary College, October 1st, the following gentlemen will be found added to the Faculty: F. S. Schoenleber, M.D., M.S.A., D.V.S. (Dean), anatomy; W. S. Harpole, M.D., pathology; T. B. Newby, V.S., materia medica and therapeutics; J. J. Millar, V.S. (Secretary), bovine medicine, obstetrics, and contagious diseases.

